# Metachronous bladder metastases from renal cell carcinoma: a case report and review of the literature

**DOI:** 10.3332/ecancer.2010.175

**Published:** 2010-03-18

**Authors:** S Melegari, G Albo, B Rocco, F Verweij, M Abbinante, O de Cobelli

**Affiliations:** Department of Urology, European Institute of Oncology, Via Ripamonti 435, 20141 Milano, Italy

## Abstract

**Introduction:**

adrenal gland, parotid gland, pharynx, eye and bladder are rare localizations of metastases of renal cell carcinoma (RCC). We report a case of metachronous RCC metastases to the bladder in a patient with a medical history of transitional cell carcinoma (TCC) of the bladder.

**Materials and methods:**

a case study and review of the relevant literature are presented.

**Results:**

during a follow-up cystoscopy examination following treatment of TCC, a single 5-mm lesion was detected and endoscopically resected. The histology of the resected sample was confirmed to be RCC, comparable to a primary kidney cancer and not recurrent TCC.

**Conclusion:**

the patient had a probability of metastases three years after nephrectomy of 62.9%. Survival rates following single metastasectomy are 60% and 38% at three and five years, respectively; metachronous diagnosis has a better prognosis than synchronous. During RCC follow-up, each lesion should be considered as a possible metastasis of RCC.

## Introduction

The incidence of kidney cancer has grown over the last few decades. According to SEER data [1], an estimated 57,760 individuals (35,430 men and 22,330 women) were diagnosed and 12,980 patients died from kidney cancer in 2009

Among the patients diagnosed with kidney and renal pelvis cancer, 58% were diagnosed when the cancer was in a localized stage; 18% were diagnosed after the cancer had involved regional lymph nodes or progressed directly beyond the primary site; 19% were diagnosed after the cancer had metastasized and for the remaining 4%, the staging information was unknown. The corresponding five-year relative survival rates were: 90.4% for localized disease; 62.3% for cancer that has progressed to regional lymph nodes involvement; 10.4% for distant metastases; and 37.5% for unstaged disease [1].

Stage and grade at diagnosis influence the prognosis of kidney and renal pelvis cancer; conversely, the outcome of the disease does not seem to be related to patient age [2].

Renal cell carcinoma (RCC), even if organ confined, can recur at any time after nephrectomy. The Mayo Clinic Scoring System can be used to predict the percentage survival rate and metastases risk on the basis of pathological stage, tumour size, regional lymph node status, nuclear grade and the presence of necrosis [3].

The RCC usually metastasizes via the haematogenous and lymphatic systems, involving the lung (50–60%), lymph node (40–60%), liver (30–40%), bone (30–40%), brain (5%), bowel (4%), pancreas (1%) and rarely the adrenal gland, parotid gland and pharynx [4]. A case of RCC ocular metastases has also been reported [5]. The bladder is a very unusual site of metastases, and in particular no cases have previously been described in Italy [6–9].

## Materials and methods

We describe the case of a 65-year-old patient affected by bladder transitional cell carcinoma (TCC), RCC and adenocarcinoma of the prostate.

Bladder TCC was diagnosed elsewhere in 1993; the patient underwent trans-urethral resection of bladder tumour (TCC stage Tx grade G2). He received no adjuvant therapy and continued to attend for regular endoscopic follow-up. The bladder tumour recurred twice: in 1997 (TCC T1G1) and in 2006 (TCC T1G2 associated with carcinoma *in situ*). He was administered bacillus Calmette-Guérin for 36 months, following the Southwest Oncology Group schedule [10].

In 2005, when he presented the for first time at the European Institute of Oncology, a cT2N0M0 prostate cancer was diagnosed (combined Gleason score 8 [4+4]; prostate specific antigen 37.7 ng/ml). An abdominal computed tomography scan revealed incidental presence of kidney cancer. Thus, the patient underwent a left radical nephrectomy and lymphadenectomy for an RCC pT3a pN0, Fuhrman grade III. The tumour was <10 cm in diameter and was necrotic. The patient subsequently underwent radiation therapy for prostate cancer (66 Gy). No evidence of prostate cancer recurrence was found in three years of regular follow-up.

In February 2009, a 5-mm lesion was found during the cystoscopy performed as a follow-up for the TCC. This lesion was endoscopically resected and histology was that of clear cell RCC. The diagnosis of RCC metastases was confirmed by comparison with the histology of the resected primary kidney cancer. Immunohistochemistry was positive for cytokeratin (ck) AE1/AE3, ck 7, ck 20, cluster of differentiation (CD10) and beta catenin, and negative for c kit, PSA and FAP (fibroblast activation protein).

## Discussion

Renal cell carcinoma rarely metastasizes to the bladder. Kagota *et al* [6] reported 30 cases in Japan; few other cases are described in the literature [8,9].

Due to the infrequency of this site for RCC metastases, the management of these lesions described in the literature varies considerably.

As far as management of single renal cell carcinoma metastases is concerned, as reported in 2005 by the Tongaonkar group [4], complete resection either by excision or radiotherapy is justified and can contribute to long-term survival.

The first metastasectomy was performed by Barney [11] in 1939 in a patient with a lung secondary tumour, who died 23 years later of coronary artery disease.

In fact, the decision to perform metastasectomy is usually made according to various prognostic criteria: the site and number of metastases [12], the completeness of resection of the primary tumour, the performance status and the disease-free interval from treatment of the primary tumour to the diagnosis of metastatic disease.

Partial cystectomy [6], endoscopic resection [6,7] and endoscopic resection followed by interleukin-2 systemic therapy [13] have all been used to treat bladder metastases.

Complete resection of the RCC single metastases is associated with five-year survival rates between 35% and 60%. Mean survival time after a single metastasectomy is 45 months [4]. The longer the disease-free interval, the longer the survival, and synchronous metastases seems to correlate to a worse prognosis than metachronous metastases.

Thus, according to the majority of authors, patients with metastatic RCC should be offered metastasectomy if the likelihood that complete resection of all sites of disease is high. Although more data are required to reach a conclusion and the curative impact of metastasectomy can be still considered uncertain, surgical intervention can provide effective palliation for symptomatic metastatic disease in such sites as bone, brain and adrenal gland [14,15].

The site of mestases can be related to the survival: patients with lung and bone metastases have a better prognosis (median survival 62 months) than those with liver and brain metastases (median survival 22 months), probably because of differences in the feasibility of a radical excision [4].

For bladder metastases, the three-year survival rate is 80% in the case of single metastases and 20% for patients with more than one site involved. About 50% of patients with multiple metastases have a life expectancy of less than one year following diagnosis; no difference was found between synchronous and metachronous metastases in terms of overall survival. The mean time to diagnosis of urinary bladder metastases is 28 months after nephrectomy (range 0 to 131 months) [16].

In our case, the diagnosis of bladder metastases was metachronous, 36 months after nephrectomy was performed. According to the Mayo Clinic scoring system for renal cancer, this patient was at high risk of metastases (62.9% at three years).

## Conclusion

During the RCC follow-up, every lesion should be considered a possible RCC metastasis even if it is not a common site for metastasis. As there is no scientific consensus in the management of these lesions, we performed a routine metastasectomy. Longer follow-up is needed to confirm the adequacy of our approach.

## References

[1] http://seer.cancer.gov/statfacts/html/kidrp.html

[2] Schlesinger-Raab A, Treiber U, Zaak D, Hölzel D, Engel J (2008). Metastatic renal cell carcinoma: results of a population-based study with 25 years follow-up. Eur J Cancer.

[3] Leibovich BC, Blute ML, Cheville JC, Lohse CM, Frank I, Kwon ED, Weaver AL, Parker AS, Zincke H (2003). Prediction of progression after radical nephrectomy for patients with clear cell renal cell carcinoma: a stratification tool for prospective clinical trials. Cancer.

[4] Thyavihally YB, Mahantshetty U, Chamarajanagar RS, Raibhattanavar SG, Tongaonkar HB (2005). Management of renal cell carcinoma with solitary metastasis. World J Surg Oncol.

[5] Mancini V, Battaglia M, Lucarelli G, Di Lorenzo V, Ditonno P, Bettocchi C, Selvaggi FP (2008). Unusual solitary metastasis of the ciliary body in renal cell carcinoma. Int J Urol.

[6] Kagota M, Irie K, Hosaka K, Takezaki T (2007). Bladder metastasis of renal cell carcinoma; a case study. Hinyokika Kiyo.

[7] Cloché Hummel MO, Houssemand G (1987). Tumeur vésicale métastatique d’un carcinoma renal à cellules claires. Journal d’Urologie.

[8] Mayer WA, Resnick MJ, Canter D, Ramchandani P, Kutikov A, Harryhill JF, Carpiniello VL, Guzzo TJ (2009). Synchronous metastatic renal cell carcinoma to the genitourinary tract: two rare case reports and review of the literature. Can J Urol.

[9] Uygur MC, Ozen HA, Sungur A, Remzi D (1994). A solitary synchronous metastasis of renal cell carcinoma to the bladder. Int Urol Nephrol.

[10] Lamm DL (2000). Preventing progression and improving survival with BCG maintenance. Eur Urol.

[11] Barney JD (2000). Adenocarcinoma of the kidney with metastasis to the lung: cured by nephrectomy and lobectomy. J Urol.

[12] Russo P, O’Brien MF (2008). Surgical intervention in patients with metastatic renal cancer: metastasectomy and cytoreductive nephrectomy. Urol Clin North Am.

[13] Shiraishi K, Mohri J, Inoue R, Kamiryo Y (2003). Metastatic renal cell carcinoma to the bladder 12 years after radical nephrectomy. Int J Urol.

[14] Wroński M, Arbit E, Russo P, Galicich JH (1996). Surgical resection of brain metastases from renal cell carcinoma. Urology.

[15] Kim SH, Brennan MF, Russo P, Burt ME, Coit DG (1998). The role of surgery in the treatment of adrenal metastasis. Cancer.

[16] Matsuo M, Koga S, Nishikido M, Noguchi M, Sakaguchi M, Nomata K, Maruta N, Hayashi T, Kanetake H (2002). Renal cell carcinoma with solitary metachronous metastasis to the urinary bladder. Urology.

